# How is modern bedside teaching structured? A video analysis of learning content, social and spatial structures

**DOI:** 10.1186/s12909-022-03855-0

**Published:** 2022-11-15

**Authors:** Anna-Lena Blaschke, Hannah P. K. Rubisch, Ann-Kathrin Schindler, Pascal O. Berberat, Martin Gartmeier

**Affiliations:** 1 TUM Medical Education Center, TUM School of Medicine, Technical University of Munich, Ismaninger Straße 22, 81675 Munich, Germany; 2 DEMEDA (Department of Medical Education), Medical Didactics and Educational Research, Medical Faculty, University of Augsburg, Universitätsstraße 2, 86159 Augsburg, Germany

**Keywords:** Teaching quality, Bedside teaching, Clinical teaching, Clinical teacher, Undergraduate medical education, Video study, Videography

## Abstract

**Background:**

Bedside teaching (BST) is an essential and traditional clinical teaching format. It has been subject to various impediments and has transformed over time. Besides a decrease in bedside time, there has also been a didactic diversification. In order to use time at the bedside effectively and understand the current design of BST, we here offer an evidence-based insight into how BST is practiced. This may serve as a basis for a refinement of its didactic design.

**Methods:**

In the current study, we investigate the interrelationships between learning content and the social as well as spatial structures of BST. To this end, we have empirically analysed almost 80 hours of video material from a total of 36 BST sessions with good interrater reliability.

**Results:**

BST lasted on average 125 min, most of which was spent in plenary and less than a third of the time at the patient’s bedside. History taking was primarily practiced at the bedside while case presentations, clinical reasoning and theoretical knowledge were largely taught away from the patient. Clinical examination took place to a similar extent in the patient’s room and in the theory room.

**Conclusions:**

Even though the filmed BSTs are not purely “bedside”, the teaching format investigated here is a typical example of undergraduate medical education. In order to maximize the teaching time available, a suitable learning space should be provided in addition to the bedside. Moreover, the clinical examination should be revised in its general sequence prior to the BST, and conscious decisions should be made regarding the social structure so as to optimize the potential of small groups and plenary sessions.

## Background

Bedside Teaching (BST) is an essential didactic format with a longstanding tradition in medical education [1–3]. It can be briefly described as “the process of active learning in the presence of a patient” ([4] p. 159). Over the past few decades, many authors have increasingly pointed out a growing number of impediments to this teaching format. Lecturers have less time [4–8], often feel poorly prepared for teaching, and have more patients to care for in total than before [6–12]. With the introduction of new technologies, the time spent at the patient’s bedside, and therefore also possibly spent teaching, has decreased, and the importance of good manual clinical examination skills seems to have declined [6, 7, 10, 13]. Poorly prepared students and the dynamic environment of everyday hospital routine have also made life more difficult for BST-lecturers [4, 5, 9, 10, 14]. Regarding the modern patient-centred approach to treatment, patients seem to reject student-teaching more often or behave uncooperatively [1, 6, 8, 15]. As a consequence of these impediments, the way BST is practiced has been adapted to better fit these changed conditions [10]. More structured approaches have been proposed [16] and implemented [12, 16–18]. Before looking at these approaches in more detail, we describe the general content of BST, as well as the observed trends towards *decline* and *diversification* of BST.

According to the relevant literature, key learning outcomes associated with BST include students’ ability to communicate with patients, take a medical history, to diagnose and perform clinical reasoning and, maybe most importantly, to model professional behaviour and humanism for future physicians [16, 19, 20]. BST gives students opportunities to acquire and refine essential clinical skills in the area of patient examination [5, 10, 11, 21, 22] and there is substantial evidence underlining the instructional value of BST [1, 10, 23]. In the present study, we address interrelationships between learning content covered in BST and the social as well as spatial structures of such courses based on detailed empirical analyses of video-recordings of BST sessions.

To substantiate why such a descriptive perspective is valuable, we focus two current trends in medical education, i.e., a decline of the time spent on BST as well as a didactic diversification in how BST is practiced. Regarding the former, many authors note that in medical curricula, less time is being dedicated to actual teaching at the bedside [24]. The reasons behind this trend are, among others, a growing reliance upon tests and technology-based diagnostics and increased amounts of time required for reviewing patient-related information at the computer [25]. An adverse effect associated with this trend is a decline of physicians’ clinical skills, e.g. regarding examination techniques [11, 13, 26]. From this point of view, a better understanding of how BST is practiced would be useful for developing fresh perspectives for its didactic design.

Another trend that can be observed lies in the didactic *diversification* of this instructional format. Traditionally, BST is integrated into ward rounds, in which the doctor, for instance, takes a patient’s history once again, demonstrates the important physical signs to his students and asks them to elicit these signs [13]. Beyond this, recent BST is becoming more and more complex and combines various didactic elements [10], especially when integrated into the undergraduate medical curriculum [27]. Examples are “reporting back: the trainee assesses the patient and reports back to the trainer” [16] or “case conferences” [16]. During the latter format, students review and discuss a patient case in small groups before doing so with the entire group and the clinical teacher. Moreover, bedside encounters are combined with elements of (off-patient) skill-lab teaching, such as providing instruction in examination techniques [28].

Both observed trends suggest that time spent at the bedside is decreasing, partly in favour of time spent in a *theory room* where, for example, case conferences or skill-lab teaching takes place [1, 27]. Furthermore, BST can also take place in the hospital hallway, as, for example, when students are briefed before entering a patient room or facts are to be discussed outside [10, 29]. Alongside the diversification of the instructional format, social structures have also become more diverse. In current BST, the plenum is not always gathered at the patient’s bedside. Often, lecturers divide BST-courses into small groups, some of which may function without direct guidance or instruction from the physician [10, 27].

In light of the described developments, we argue that medical education research is challenged to attain more detailed, evidence-based insights into the *structure* and *content* of current BST. Such insights are helpful in understanding the challenges and devising concepts for its further development. In adopting this aim in the present study, our first research focus is upon the spatial and social structures in BST sessions as well as the learning content covered. As a basis for our study, we draw upon the typical learning objectives of BST reported in the literature [9, 30, 31]. These are clinical skills, such as history taking, clinical examination, case presentation, clinical reasoning and theoretical knowledge. Additional objectives reported in the literature are growing into the role of a physician, humanism, and learning to establish a good physician-patient interaction [6, 20]. In contrast to these learning objectives, however, it is difficult to assign these learning objectives to specific instructional elements of BST sessions.

Second, we investigate interrelations between learning content and the spatial as well as social structures of BST. More specifically, we investigate differences regarding learning content, depending on the location where the learning takes place (patient vs. theory room), and the social form in which the learning occurs (plenary vs. group). We thus address the following research questions (RQs) in the present study:

- RQ1: Which spatial and social structures and learning content can be identified in BST sessions?
- RQ2: Are there differences in content depending on a) the location (patient vs. theory room) and b) the social structure (plenary vs. group)?

## Method

### Bedside teaching sessions

We video-recorded BST-sessions in clinical medical education at Klinikum Rechts der Isar, a German university hospital. Each course was attended by around 6 students, mostly in their second clinical year (4th year overall) and scheduled for 180 minutes. In the local medical curriculum, a total of 360 such courses are offered per semester over a time span of 12 weeks. Each student takes part in a total of 8 BST-sessions in internal medicine, 2 in neurology and 2 in orthopaedics. In the local curriculum, the BSTs build on examination courses in the first clinical year. They are accompanied by in-depth lectures in the respective subjects in the second clinical year and prepare students for the block placements in their third clinical year. The BST-sessions in our study featured traditional patient contact, but were enriched by off-patient seminar-style sequences in which theoretical aspects and practical skills were in focus. The clinical teachers were themselves doctors in the respective specialist areas.

Although organizational and hardware constraints limited our choice of BSTs, the sessions from three specialties - internal medicine, neurology and orthopaedics - provide a broad picture of BST. Their somewhat different character is described in the following:

#### Neurology

In neurology, the emphasis was on repeating basic knowledge and skills as well as consolidating, deepening and appropriately applying the clinical examination techniques. To this end, students were supervised by the same doctor on both consecutive dates. First, the clinical-neurological examination was repeated and practiced again on each other before the students performed it independently on the patient. In the second session, the focus shifted to the skills of case presentation, diagnosis, clinical reasoning, and therapy planning. These skills and the necessary knowledge were practiced involving one or more patients. Often, the group was divided into smaller groups or pairs for this purpose.

#### Internal medicine

The procedure in internal medicine was basically similar to that in neurology, whereby the groups of students were each accompanied by their respective clinical teacher for only one afternoon. The specialist areas of internal medicine comprised two sessions each: Cardiology/Pulmonology, Haemato-Oncology, Nephrology/Rheumatology/Toxicology and Gastroenterology.

#### Orthopaedics

In the orthopaedic wards, most of the patients had had surgery. Hence, in order not to interfere with the healing process of the surgical wounds, the focus of the orthopaedics encounters was on the areas of anamnesis, case presentation, diagnosis, clinical reasoning and therapy planning. In these sessions, students occasionally learned specific examination techniques, which they practiced on each other. Also, the two-consecutive orthopaedics-seminars built on each other since they were led by the same doctor.

### Sample, recruitment and ethical considerations

We filmed a total of 36 BST-sessions, 12 each from internal medicine, orthopaedics and neurology. The selection of BSTs took place at least two weeks before the respective session. For the sake of the patients” well-being, BSTs taking place in the intensive care unit or the outpatient area were excluded from the selection process. One week prior to the filming, the clinical teacher was contacted to discuss possible changes in the work schedule and to answer questions. The doctor in charge selected suitable patients on the ward and asked them for their consent to participate in the bedside teaching as well as in the study. The students on the courses were contacted in advance via e-mail and had the possibility to change to another course if they did not wish to be filmed.

The 36 BST-sessions filmed had a cumulative length of 78.43 hours. A total of 24 different clinical teachers led the courses and 259 students and 84 patients took part. 7 students switched courses in order not to be filmed. 47 of the 84 patients were featured in our video material (the other patients were visited by subgroups of students, who were not accompanied by a cameraperson from our team). Information sheets and consent forms were prepared. At the beginning of each BST-session, a member of the research team was present to give information about the study and answer questions once again. We obtained full consent prior to any filming. Anonymity of all participants was assured by self-selected pseudonyms for clinical teachers (3 characters for the identification of the lecturers, who held two seminars) and the avoidance of clear names for students and patients. Our study was approved by the local ethics committee (Application code 360/18S).

### Data collection: videography

The main study data collection took place in winter 2018/19. In summer 2018, we had already conducted a pilot-study, in which we had filmed three BST-sessions in order to check the camera set-up and to obtain data for training purposes during later coding (see below). In the following, unless otherwise indicated, all data refer to the main study.

**Fig. 1:**
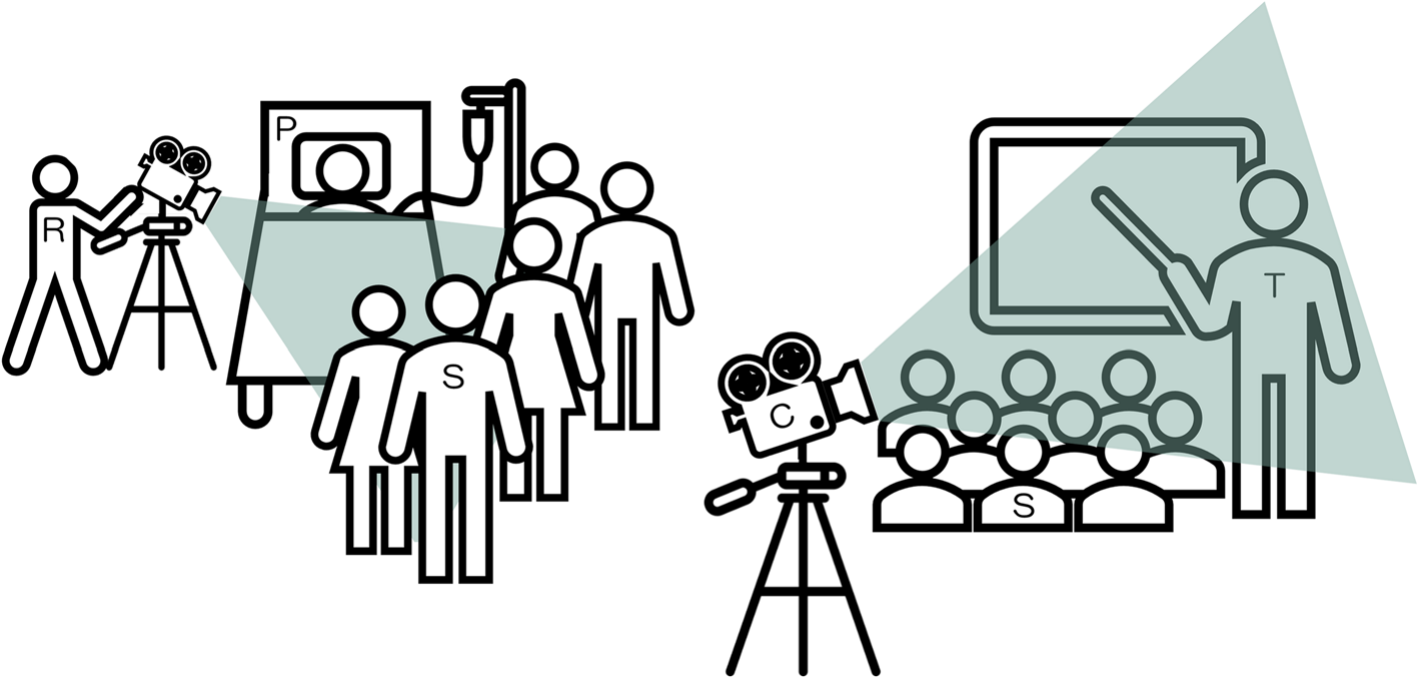
Camera setup Exemplary Camera setups in the patient room (left) and seminar room (right). C = Camera, *P* = Patient, R = Researcher, S = Student, T = Medical Teacher. Components of this graphic are provided unrestricted and copyright-free by Microsoft

We used a video camera with tripod and directional microphone to record the BST sessions (see Fig. 1). In order to ensure comparability of the videos, we followed a set of recording guidelines: We started the video recording only after having obtained consent from all participants. The recording was not paused or stopped until the lecturer had finished the BST-session. In general, we filmed in overview perspective so that the lecturer, students and other objects (e.g. X-rays, computer screens) were easily visible. The patient was filmed in a way that left the face invisible on video (see Fig. 1: Camera setup in the patient room). At the beginning of the seminar, a student was silently designated, whom the cameraperson followed, e.g. in phases of small-group work. If several patients were simultaneously examined by the students, the camera remained with the one pre-designated student and did not switch between groups. The cameraperson took care, as far as possible, not to disturb the teaching process.

### Data collection: online evaluation and questionnaires

In addition to the filmed BST-sessions, our research team had access to the student evaluations of all 360 bedside teaching courses (German educational grades from 1 to 6, with 1 being the best possible). The demographic data of the students and lecturers were collected by means of a questionnaire at the end of the course. We also used a Likert scale to assess how much the presence of the camera affected the course of the BST session and the behavior of the students (Likert scale from 1 to 6, with 1 being “not at all” and 6 “very much”).

### Processing the video data: categorial scheme and coding

**Fig. 2:**
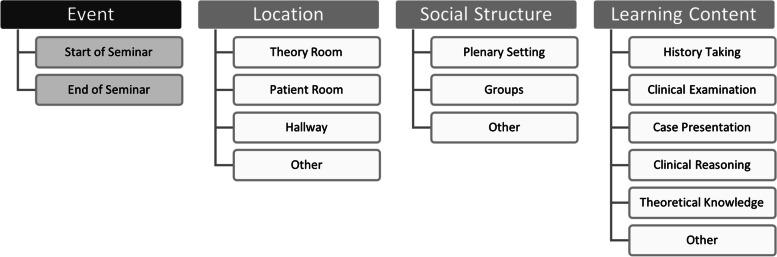
Categorial System Single time markers were set for the start and end of the seminar. “Location”, “Social Structure” and “Learning Content” were coded for time spans independent of each other and form an exhaustive-disjunctive category system

We coded the video material regarding the *location, social structure* and *learning content* (see Fig. 2). The underlying coding scheme was literature-based and had an exhaustive-disjunctive character [32]. Initially defined categories were revised several times in an iterative process with the help of the video material from the preliminary study. The locations typically used in a BST (theory room, patient room and hallway) have already been outlined above. The *patient room* is defined as the room assigned to the patient for the hospital stay with their bed, the *theory room* is defined by its function (knowledge exchange in a room that is not a patient room) and can be, for example, the doctor’s room, an empty patients’ kitchen or a special seminar room. We summarised the hallway, the corridor and the staircase under the code *hallway*. In addition, we added the code *other* for places that did not fit any of the above descriptions.

In order to describe the *social structure* more precisely, we distinguish between *plenary setting* and *groups*, depending on whether all students were working together, or were divided into groups. Since individual work was very rare, we have included it in the code *other.* Furthermore, all social structures that do not fit into either of the other two codes fall under *other*. In our study, the plenary consisted of a median of 6 students (*IQR* 5-10). When the plenum was divided into groups, there was a median of 2 groups (*IQR* 2-3). We relied upon pertinent literature [11, 18, 28] documenting the *learning content* (see Fig. 2) typically featured in bedside seminars. We focused on readily observable skills and knowledge, as attitudes and professional behaviour such as communication and teamwork tend to be taught in passing rather than explicitly.

Two researchers (AB and HR) coded the video material. Before starting the playback of the video, the initial *location*, *social structure* and *learning content* were determined. In the initial training phase, a video from the preliminary study was coded by both raters, the results were visualized, compared and the category system was further refined in this process. Afterwards, the two raters coded the remaining two videos and discussed deviations. Since the interrater reliability was satisfactory, we proceeded to the main study.

In the main study we calculated the interrater reliability based on four videos with a total duration of just over 10 hours. We achieved “almost perfect” [32] interrater reliability for *location* (0.97), *social structure* (0.98) and *learning content* (0.83). The interrater reliability was calculated on the basis of 37,418 one-second intervals, which were generated after coding.

### Statistical analyses

We conducted both descriptive and inferential statistical analyses, using IBM SPSS for Windows (version 26.0). The significance threshold was set to *p* < 0.05 and in cases of multiple testing (RQ2), we applied the Bonferroni correction. For 24 tests (see Table 1 and 2), the adjusted significance value was p* < .002. In the following, discrete characteristics are described using median (*MD*), range and interquartile range (*IQR*). We focus on absolute and relative durations of the video codes location, social structure and learning content. The respective data were determined as continuous, but not normally distributed, and therefore two-sided Mann-Whitney U tests were used to test for differences in the central tendency in teaching in the patient and theory room (RQ2a) as well as for the social structures (RQ2b).

**Table 1:** Learning content in patient- and theory room

Learning Content	Predo-minant Location	Theory Room			Patient Room			Mann-Whitney U		
		MD [min]	IQR [min]	Range [min]	MD [min]	IQR [min]	Range [min]	*p*-values	U	Z
History Taking	Patient Room	0.00 *(0.00%)*	0.00-0.13 *(0.00 -0.09%)*	0.00-20.48 *(0.00 -20.46%)*	10.81 *(41.03%)*	4.90-18.15 *(18.21 -73.37%)*	0.00-41.12 *(0.00 -91.23%)*	*p* < .001* p < .001*	85.00 *38.50*	−6.56 *−7.02*
Clinical Examination		3.48 *(5.21%)*	0.00-31.79 *(0.00 -43.82%)*	0.00-115.37 *(0.00 -88.97%)*	13.47 *(37.26%)*	0.00-36.23 *(0 -69.28%)*	0.00-69.55 *(0.00 -84.46%)*	*p* = .842 *p = .158*	630.00 *508.50*	−.21 *−1.42*
Case Presentation	Theory Room	10.88 *(17.75%)*	5.23-22.27 *(6.20 -36.79%)*	0.00-77.05 *(0.00 -72.74%)*	0.00 *(0.00%)*	0.00-0.00 *(0.00 -0.00%)*	0.00-15.32 *(0.00 -23.27%)*	*p* < .001* p < .001*	81.00 *89.50*	−6.61 *−6.42*
Clinical Reasoning	Theory Room	13.14 *(25.08%)*	7.30-35.20 *(14.13 -36.98%)*	0.00-78.60 *(0.00 -81.91%)*	0.00 *(0.00%)*	0.00-0.00 *(0.00 -0.00%)*	0.00-8.15 *(0.00 -10.81%)*	*p* < .001* p < .001*	46.00 *38.50*	−7.07 *−7.08*
Theoretical Knowledge	Theory Room	5.52 *(6.16%)*	0.29-13.3 *(0.28 -16.54%)*	0.00-34.67 *(0.00 -45.41%)*	0.00 *(0.00%)*	0.00-0.00 *(0.00 -0.00%)*	0.00-6.83 *(0.00 -6.06%)*	*p* < .001* p < .001*	195.50 *184.00*	−5.69 *−5.71*
Other		8.02 *(10.64%)*	3.99-12.33 *(7.60 -17.05%)*	0.48-49.82 *(0.73 -47.46%)*	3.65 *(12.51%)*	2.07-5.79 *(8.77 -16.30%)*	0.00-18.17 *(3.33 -32.17%)*	*p* < .001* *p = .381*	314.50 *553.00*	−3.76 *−.89*

The star (*) indicates significant differences in the central tendency using a two-sided Mann-Whitney U test after correction for multiple testing (p* < .002)

*MD* Median, *IQR* Inter-Quartile-Range

**Table 2:** Learning Content in Plenary Setting and Groups

Learning Content	Predom. Social Structure	Plenary Setting			Groups			Mann-Whitney U		
		MD [min]	IQR [min]	Range [min]	MD [min]	IQR [min]	Range [min]	p-values	U	Z
History Taking		0.13 *(0.09%)*	0.00-6.30 *(0.00 -6.98%)*	0.00-29.17 *(0.00 -24.06%)*	7.43 *(30.82%)*	0.00-12.37 *(12.79 -42.2%)*	0.00-29.70 *(0.00 -83.79%)*	*p* = .016 p < .001*	442.00 *123.00*	− 2.40 *−5.25*
Clinical Examination		16.45 *(16.83%)*	2.62-44.86 *(2.88 -50.23%)*	0.00-137.93 *(0.00 -80.81%)*	5.97 *(31.6%)*	0.00-17.28 *(0.00 -46.30%)*	0.00-40.08 *(0.00 -100%)*	*p* = .003 *p = .855*	395.00 *490.00*	−2.90 *−.19*
Case Presentation	Plenary Setting	14.13 *(14.07%)*	6.19-21.36 *(5.34 -30.52%)*	0.70-66.95 *(1.10 -62.03%)*	0.00 *(0.00%)*	0.00-0.96 *(0.00 -4.38%)*	0.00-12.05 *(0.00 -25.48%)*	*p* < .001* p < .001*	71.00 *124.00*	−6.68 *− 5.21*
Clinical Reasoning	Plenary Setting	17.53 *(20.49%)*	9.18-33.16 *(9.78 -32.79%)*	0.00-78.60 *(0.00 -88.16%)*	0.00 *(0.00%)*	0.00-0.00 *(0.00%-0,79%)*	0.00-29.22 *(0.00 -36.98%)*	*p* < .001* p < .001*	60.00 *77.00*	−6.85 *− 5.88*
Theoretical Knowledge	Plenary Setting	6.46 *(5.22%)*	0.44-12.64 *(0.49 -16.81%)*	0.00-34.67 *(0.00 -32.36%)*	0.00 *(0.00%)*	0.00-0.00 *(0.00 -0.00%)*	0.00-17.03 *(0.00 -23.47%)*	*p* < .001* p < .001*	211.00 *203.00*	−5.41 *− 4.36*
Other		13.45 *(15.43%)*	8,43-21,10 *(9.52 -23.04%)*	1.25-51.60 *(1.86 -35.57%)*	5.48 *(26.89%)*	0.02-11.63 *(15.93 -31.11%)*	0.00-46.57 *(0.00 -100%)*	*p* < .001* *p = .003*	312.00 *287.00*	−3.79 *− 2.94*

The star (*) indicates significant differences in the central tendency using a two-sided Mann-Whitney U test after correction for multiple testing (p* < .002)

*MD* Median, *IQR* Inter-Quartile-Range

### Influence of videography on the filmed bedside seminars

In order to evaluate the representativeness of the study data and to estimate how much the camera interfered with the course of the seminars, we used online evaluation data of all 360 courses and our questionnaire data.

We compared the general online student evaluations (German educational grades from 1 to 6, with 1 being the best possible) of the 36 courses with camera attendance with the 313 courses that were not included in the study and could not find any significant difference with a t-test (*MN*_*36*_ = 1.48; *SD*_*36*_ = .67; *MN*_*313*_ = 1.55; *SD*_*313*_ = .72; *p* = .572). For a further 11 courses (out of the 360 courses in total), which were also not part of the video study, no evaluation data were available.

The influence of the camera on the course of the BST-session and the student’s behaviour was rated on a Likert scale (Likert scale from 1 to 6, with 1 being “not [influenced] at all” and 6 “very much” [influenced]) by the physicians. The rating doctors (*N* = 36) found little influence on either the course of the BST-session (*MN* = 1.67, SD = .986) or the students’ behaviour (*MN* = 2.00, *SD* = 1.12).

## Results

### RQ1: spatial and social structure, learning content

**Fig. 3:**
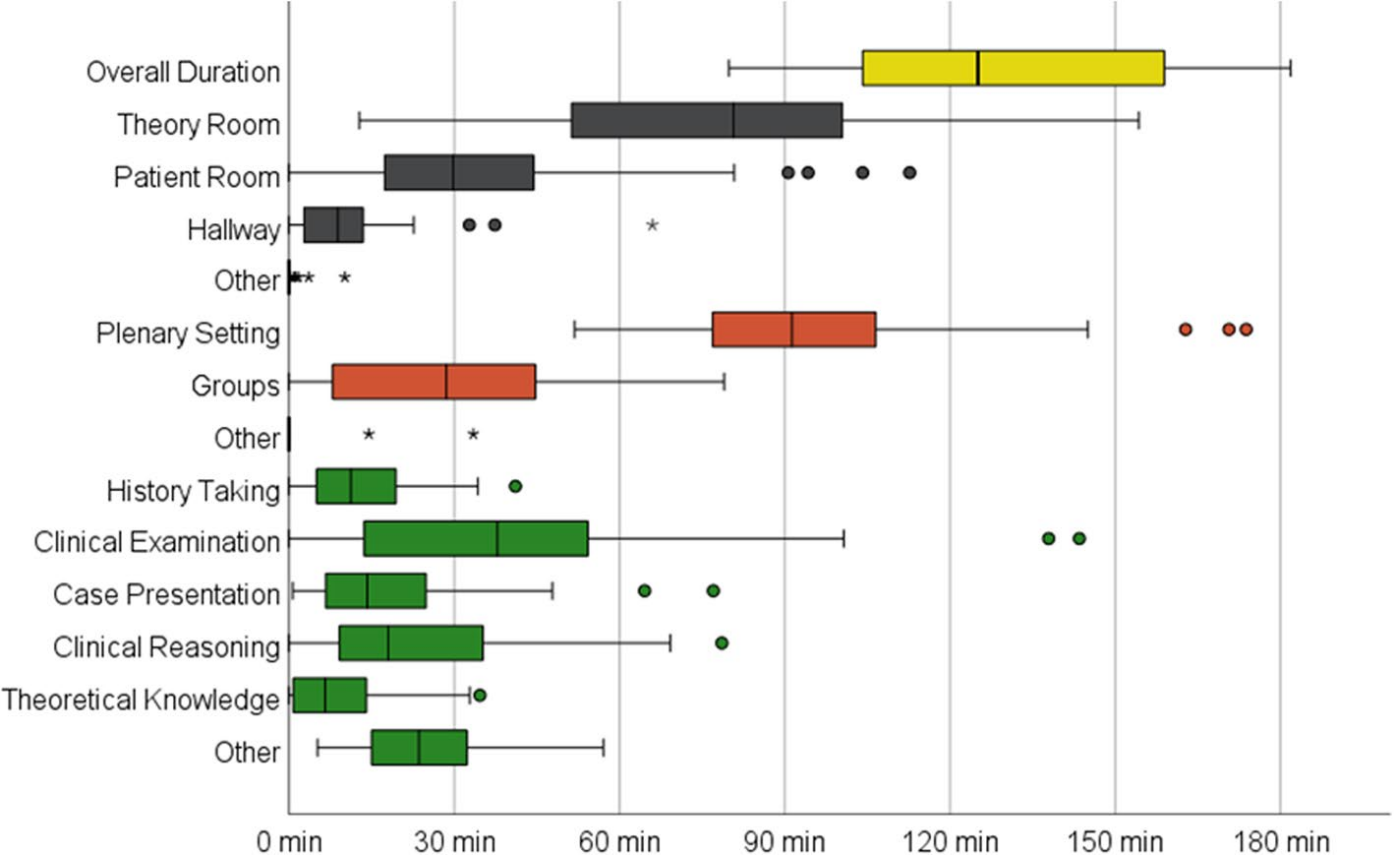
Boxplot over the total duration of individual codes Boxplots showing the overall duration (yellow), location (black), social structure (orange) and learning content (green)

The 36 filmed BST-sessions had a median duration of 125 minutes, the shortest seminar lasted 80 and the longest 182 minutes, with an IQR from 104 to 160 minutes (yellow box in Fig. 3). In general, the data show a very heterogeneous picture of the BST-sessions, which can be seen in the strong scattering of the data (e.g. outliers and long whiskers) and the large interquartile ranges.

#### Spatial and social structure

Regarding location (black boxes Fig. 3) more than twice as much teaching occurred in the *theory room* (*MD* = 80 min, *IQR* 51-101 min) compared to the *patient room* (*MD* = 30 min, *IQR* 17-44 min). The *hallway* plays a minor role (*MD* = 9 min, *IQR =* 3-14 min). The code *other* for a location was almost never used. Teaching in a plenary setting was the predominant social structure (orange boxes in Fig. 3) with a median duration of 91 minutes (*IQR* 77-107 min), the remaining time was almost always spent in groups (*MD* 29 min, *IQR* 6-46 min). 76.20% of the group time was spent in the patient room and 81.30% of the plenary time was spent in the theory room.

#### Learning content

In 81.4% of the time, the learning content could be assigned to a specific code in our category system (therefore not *other*). In detail, the largest amount of time in the seminars was dedicated to *clinical examination (MN* = 38 min, *IQR* = 14-55 min). Then followed *clinical reasoning* (*MN* = 18 min, *IQR* = 9-35 min), *case presentation* (*MN* = 14 min, *IQR* = 7-26 min) and *history taking* (*MN* = 11 min, *IQR* = 5-20 min). The smallest share of time was spent on discussing *theoretical knowledge* (*MN* = 7 min, *IQR* = 1-14 min). The code *other* lasted a median of 24 minutes, with an interquartile range from 15 to 34 minutes. During this time, for example, organizational issues were clarified, or the observed group waited until the plenum gathered again.

### RQ2a: different focus in the patient and theory room

**Fig. 4:**
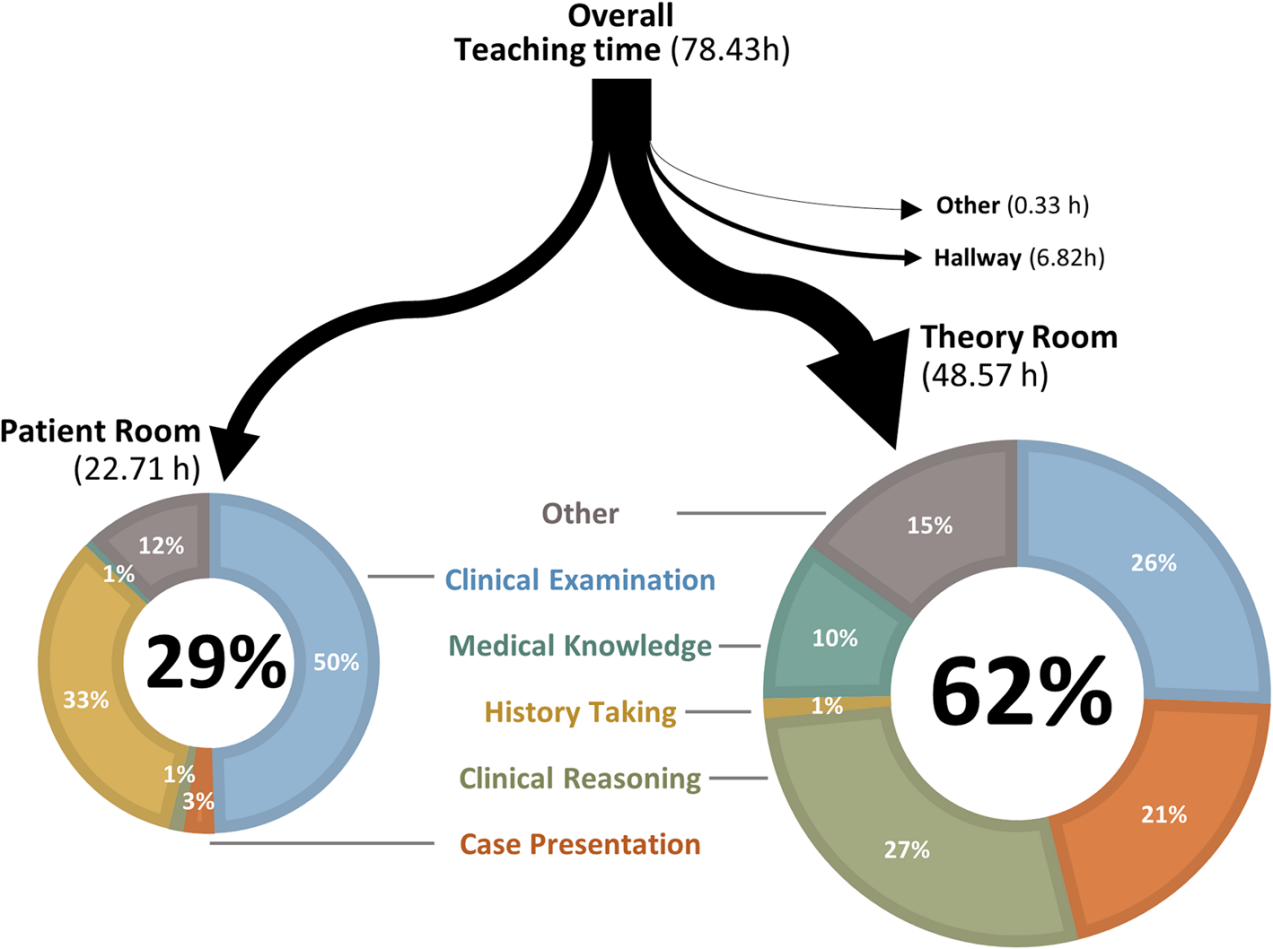
Learning content in patient- and theory room The figure was created on the basis of the totals of our overall data. The circle diagrams’ areas and the arrows widths are proportional to the amount of teaching time they represent. For reasons of clarity, we do not present the learning content for the locations “Other” and “Hallway”

Out of the total 78.43 hours of seminar, 48.57 hours took place in the theory room and 22.71 hours in the patient room. Figure 4 displays the distribution of the learning content.

Due to the large differences in seminar length (from 80 to 182 minutes), we report below not only the easily understandable, absolute values, but also the percentages in duration of the entire seminar (see Table 1).

The differing focus of the seminar room and the patient room was evident in four of the six codes for learning content: *Case presentation*, *clinical reasoning* and *theoretical knowledge* were predominantly taught in the seminar room. The teaching and practice of *history taking*, in contrast, occurred almost exclusively in the patient’s room. In the median, the *clinical examination* was taught more in the patient’s room, but this difference is not significant. This finding must be interpreted in the context of the large scatter of the data. In absolute terms, the code *other* occurred more often in the theory room, but there was no significant difference in the relative durations.

### RQ2b: different focus in plenary setting and groups

Finally, we analysed which learning content was taught using which social structure (see Table 2). Some learning content was taught significantly more in plenary (*case presentation*, *clinical reasoning* and *theoretical knowledge*). The median value for *history taking* indicates it was taught longer in groups, but this correlation is only significant when looking at the relative proportions. Clinical examination was taught more in plenary sessions on average. This distributional difference is not significant. In absolute terms, the code *other* occurred more often in the plenary setting, but there was no significant difference in the relative durations.

## Discussion

By examining almost 80 hours of bedside teaching video material, we have gained insights into both the structure and content, as well as into the interrelationships of these very parameters.

The courses studied were first of all very heterogeneous. On average, they were a little over 2 hours long, of which less than a third of the time was spent in the patient room. Three quarters of the time was spent in plenary, mostly in the theory room, and one quarter of the time was spent in groups, mostly in the patient room. On average, history taking took about 14 minutes and clinical examination about 40 minutes. The discussion of the patient’s case additionally took about the same amount of time (case presentation: 18 minutes, clinical reasoning 24 minutes). The teaching of theoretical knowledge played a minor role at just under 9 minutes.

In comparing BST filmed in our study with other findings on BST, it is important to be aware of the differences between BST for undergraduate medical students and postgraduate learners, during or in addition to patient care, whether spontaneously or planned. In our setting, we are looking at BST in undergraduate teaching that is planned and integrated into a student’s schedule.

With a median of 125 min, the BST lessons analysed here are longer than most traditional BST encounters. Reported times vary between a quarter and half an hour per patient [33–35], while whole BST ward rounds are estimated at about one and a half hours [36]. Startlingly little of this time is reported to be actually spent at the bedside, often ranging from about 10 to 20% [6, 11, 13, 17, 20, 36]. Physical examination was reportedly taught in just over one third of the teaching sessions [36]. In our sample clinical examination was taught in all but three seminars and accounted for almost a quarter of the teaching time. In 35 of 36 seminars, students saw at least one patient and the total teaching time at the patient’s bedside accounted for 29% of the total course time filmed.

As was expected, the teaching of certain skills primarily took place in the theory room (case presentation and clinical reasoning), while other skills were mainly taught in the patient room (history taking). Clinical examination was not taught significantly more often in one room than in the other. The medical teachers also adapted the social structure to the respective learning content - while history taking and clinical examination were taught both in groups and in plenary, skills that are more distant from the patient were taught predominantly in the plenary setting.

Based on these results, critical questions could be raised whether the teaching filmed is actually BST, since it goes beyond teaching in the presence of the patient alone and, as the results of RQ1 show, only less than a third of the time was spent at the patient’s bedside.

On the one hand, Gonzalo ([37] p. 794) argues that in order to assign the label BST, the following should occur in the presence of the patient “(1) case presentation/history, (2) performance of at least one physical exam skill, and (3) discussion of the patient’s daily plan of care” in order to actually qualify as BST. As the results of RQ2a show, case presentation and clinical reasoning almost never take place in the presence of the patient.

On the other hand, the teaching filmed is the format within the education of undergraduate medical students in Germany that comes closest to BST. Other authors [27] describe formats similar to our sample as BST. While the clinical examination practiced outside the patient’s room is more of a repetition of known and possibly new examination techniques or serves the doctor’s assessment of how well the students can conduct the examination, it makes sense with undergraduate medical students not to perform case presentation and clinical reasoning in the patient’s room. In a protected theory room, it is easier to ask questions and make mistakes, and supporting materials such as laboratory findings or x-rays can be accessed directly. Hence, our results underline the didactic value of skills teaching taking place prior to BST in order to avoid having to practice the clinical examination on each other during BST. It is also noticeable that the courses, which were planned to be 3 hours long, only lasted about 2 hours. Various reasons for this are conceivable (concentration time of the students, workload of the physicians), which might have to be evaluated and taken into account when planning BST courses.

While physicians seem to be finding it increasingly difficult to incorporate clinical investigation into ward rounds [1, 6, 13, 38], structured seminars offer a clear advantage here [16]. Teaching special examination techniques takes time and is not always possible when efficiency is a priority in real-world patient care. Here, new forms of BST offer the enormous advantage that the physician is given time off during this period and does not have to worry about patient care. In our sample, the clinical examination was explained, demonstrated and practiced in the theory room to a greater extent than it was actually done in presence of the patient. This ensures that all students can learn examination techniques without unduly stressing the patient.

The teachers’ approach is based on various, subject-specific reasons: In orthopaedics, only patients who had already undergone surgery could be examined on the ward. To ensure optimal healing, the joints were not allowed to be re-examined by students. Although all students had already mastered the basics of the examination techniques, the physicians often used times in the theory room to repeat what was already known and to explain special techniques that were new to the students.

For many aspects of clinical practice related to the doctor-patient interaction, to physician professionalism and humanism, it seems very difficult to create adequate off-bed substitutes [4, 8, 11, 19, 30, 39]. In the same sense, physical findings can only insufficiently be replaced by images, and the sensation of palpating an effusion in a joint or triggering a pathological reflex cannot be simulated authentically, by even the best models. Students who see patients with real diseases and examine them themselves form so-called illness scripts [1, 20, 40], which comprise non-verbalizable, sometimes very subtle symptoms and findings. Only then, together with the relevant knowledge about the disease, can students develop a complete picture (“illness script”) of the disease. Therefore, part of the teaching will always have to take place at the bedside, as there are circumstances, symptoms and matters that cannot be adequately described or simulated.

On the other hand, many other learning effects so far attributed to teaching at the patient’s bedside can possibly be achieved and practiced very well in seminar rooms, as has happened in many courses in our sample. This makes BST a good example of authentic learning, which is rooted in constructivist theory. According to this theory, active and problem-oriented engagement with issues and subjects provides the best way to learn. Authentic learning includes both activities in a real-world context, such as a clinical examination, and those with a strong connection to reality, such as case discussions in the theory room, not directly related to patient care. The value of this teaching method lies in the fact that students learn to think like a member of their discipline (in our case doctors) [41]. Stein et al. [42] argue that authentic learning activities can be useful in filling the gap between lecture content and the skills needed for professional practice.

Our observations make us confident that despite the new challenges that modern developments in hospital life poses to BST, new didactic approaches can be used to find ways to continue providing excellent teaching at the patient’s bedside. For these approaches, it is important that there is precise content alignment with previous course formats so that BST can actually occur at the bedside as students master the examination techniques from previous seminars and the physician can also fully rely on the students’ expertise.

The data collection was based on 36 bedside seminars, we processed 78.43 h of video material. We argue that this is a solid data basis for answering the research questions posed here. For comparison, Monrouxe [35] describes 6 bedside teaching encounters with a total duration of 112 min recorded in the frame of a comparable study. Rees [34] analysed 7 bedside teaching sessions with 4 doctors and 2 students with a total duration of 193 min. Rizan [33] examined 12 bedside teaching encounters with 12 patients, 4 doctors and 4 students (209 min). Since all the above-mentioned studies analyse their data in a low inference manner (e.g. conversation analysis), however, the smaller data sets were sufficient there.

With our video data, a genuine qualitative content analysis would have been possible alongside the quantitative analysis. Using a mixed methods approach, as was done in other video-based studies [43] might have provided deeper insights into process-related aspects of BST. However, due to limited resources, this would have required us to reduce the size of our sample; we hold the sample size to be a strength of our study.

Despite the fact that we hypothesized that our camera would potentially disturb clinical teachers and students, we found that the students did not evaluate the seminars in which the camera was present as significantly different than the other seminars (*p* = .572). With an average of 1.67 points on the Likert scale (1-6), the lecturers’ assessment was between “1 = not at all” and “2 = slightly” [44] as to how much influence the camera had on the course of the seminar. It should be taken into account that the question refers to the entire seminar, and thus also to the beginning before the filming, in which a member of the research team explained the study. Interestingly, the lecturers evaluated the influence on the behaviour of the students with 2.00 (“slightly”) [44] on average, thus saw a greater change in behavior than in the course of the seminar. These findings also reflect the assessments of other video studies in medical education [45].

Although we are convinced that choosing videography as our research method is a great strength of the study, it can also be seen as a limitation. Participation in the study was voluntary for all medical teachers, so it can be assumed that we were more likely to accompany doctors interested in teaching and research and that patients and doctors would have behaved differently in the context of the camera being present. However, our control instruments (comparison with the non-filmed seminars and the questionnaires) showed that there were no major changes due to the camera.

Although drawing upon video recordings is not new in medical education research, it is only in the last few years that technical developments have led to the development of refined techniques for video analysis. In contrast to earlier transcript-based approaches [33], any aspect of behavior can in this way be directly observed and coded on video. In our opinion, videography is a demanding and labor-intensive method in its implementation, but if it is possible to meet the methodological requirements of scientific rigour and reproducibility, it is a rewarding source of information. As we were also able to find a satisfactory answer to potential ethical concerns, we believe that videography is a highly suitable method for exploring the content and structure of BST seminars.

## Conclusions

With the help of videography, we have gained systematic insights into bedside teaching as it is currently practiced in undergraduate medical education. This traditional and powerful teaching format has been subject to various impediments and has changed over time. On the basis of the insights gained through out study, we are confident that through adaptations and didactic innovations, a versatile BST format can be created that makes excellent teaching possible even in these framework conditions. Some key conclusions can be drawn from our analysis: First, in terms of location, a suitable room should be provided for theory so that teaching does not have to take place in the corridor. Second, in order to maximise time in the patient room, clinical examination should be intensively rehearsed by the students prior to the courses, rather than having to be practiced in the theory room. Opportunities for preparation could be provided by peer-teaching formats or a blended-learning approach, which would facilitate the study of the theory and process of a physical examination directly before the actual BST. In addition, the doctor should have an overview of the acquired level of knowledge in order to be able to entrust students directly with the examination of patients. In terms of social form, dividing the plenary into groups allows students to encounter and examine more patients. As the accompanying doctor can only give important feedback and supervision to one group at a time, a conscious decision should be made about the social form that best serves the learning objectives. Regarding the aspect of general organization, the planned three hours were too long in our sample. Reasons for the actual decrease should be evaluated; if necessary, a division into several, shorter units would make sense. Regarding further research, we recommend that our primarily descriptive, video-based approach should be extended and complemented, e.g. by qualitative studies on how structural aspects affect the way in which teachers and students interact in context of BST.

## Data Availability

All data (except the original video recordings) and materials are available upon direct request to the corresponding author: annalena.blaschke@tum.de.

## Abbreviations

BST: Bedside teaching
IQR: Inter Quartile Range
MD: Median
MN: Mean
SD: Standard Deviation
TUM: Technical University of Munich
